# Reply to comment on Treatment-related toxicity, utility and patient-reported outcomes of head and neck cancer patients treated with proton therapy: A longitudinal study

**DOI:** 10.1016/j.ctro.2025.101013

**Published:** 2025-07-14

**Authors:** Yi Hsuan Chen, Michiel Kroesen, Mischa S. Hoogeman, Matthijs M. Versteegh, Carin A. Uyl-de Groot, Hedwig M. Blommestein

**Affiliations:** a Erasmus School of Health Policy & Management, Erasmus University Rotterdam, Rotterdam, the Netherlands; b Department of Medical Physics and Informatics, HollandPTC, Delft, the Netherlands; c Department of Radiotherapy, Erasmus MC Cancer Institute, University Medical Center Rotterdam Rotterdam, the Netherlands; d Institute for Medical Technology Assessment, Erasmus University Rotterdam, Burgemeester Oudlaan 50, 3062 PA Rotterdam, the Netherlands

**Keywords:** Proton therapy, Head and neck cancer, Quality of life, Health-related quality of life, Radiotherapy-related toxicity

We thank Dr. Somay and colleagues for their thoughtful reflections on our recently published study evaluating treatment-related toxicity, utility, and patient-reported outcomes in head and neck cancer (HNC) patients treated with proton therapy (PT). Their comments raise important considerations that we would like to elaborate on.

As noted, our study focused on patient-reported outcomes (PROs) to capture the subjective experience of treatment, using validated instruments such as the EORTC QLQ-C30, QLQ-H&N35, and EQ-5D-5L. In addition to PROs, we also collected physician-reported toxicity data, with radiation-related toxicities assessed and graded by radiation oncologists according to the Common Terminology Criteria for Adverse Events (CTCAEv5.0). While we agree that incorporating additional objective assessments, such as salivary gland function tests or instrumental swallowing evaluations, could provide further insight into the toxicity burden, we believe that the combination of clinician-reported outcomes (ClinROs) and PROs offers a comprehensive and clinically meaningful perspective on patient toxicity. This dual approach captures both the external clinical manifestations and the personal, subjective experience of treatment side effects. Although we did not perform a formal correlation analysis between ClinROs and PROs in this study, future work exploring these relationships could provide valuable insight into the extent to which physician and patient perspectives align or diverge [1,2]. This patient-centered perspective is increasingly recognized as essential in the evaluation of treatment value.

Importantly, the EQ-5D-5L used in our study generates utility scores through an indirect valuation approach, wherein responses across five health domains are converted into a single summary index using a country-specific value set derived from general population preferences [3]. This allows for comparability across indications and supports use in economic evaluations.

We acknowledge the importance of late toxicities such as osteoradionecrosis (ORN), which are likely to manifest beyond our current two-year follow-up period [4]. The comparative incidence of ORN following proton versus photon therapy remains a scientifically important but currently inconclusive question. Recent evidence has reported higher ORN rates with photon therapy, approaching 10 %, although comparisons are complicated by differences in ORN definitions and grading criteria across studies [5]. A recent systematic review reported lower pooled ORN rates of approximately 5 % for grade 1 or higher and 1 % for grade 3 or higher [6], further highlighting the variability in reported incidence. Future research with larger patient populations will be essential to investigate this issue in more depth. This analysis, however, is beyond the scope of the present study. We also appreciate the emphasis on tumor heterogeneity and the varying anatomical and functional implications of different HNC subsites. While our cohort includes patients with various tumor locations, most had not undergone prior surgery, and the majority received radiotherapy with or without chemotherapy. As such, the toxicities observed are primarily attributable to the treatment itself rather than tumor-related effects. From this perspective, our population may be considered relatively homogeneous with respect to evaluating treatment-related toxicity. Nonetheless, we recognize that certain anatomical subsites, such as the oral cavity or oropharynx, may carry a higher risk for specific complications like dental toxicity or ORN due to their proximity to critical structures [7,8]. Future studies with larger, multicenter datasets may enable more detailed subsite-specific analyses.

In summary, we welcome the thoughtful commentary and agree that future research would benefit from incorporating objective toxicity assessments, longer follow-up to capture late effects, and stratified analyses by tumor subsite in larger populations. These efforts will further enrich our understanding of the clinical and patient-centered perspective on toxicities in HNC.

## Declaration of competing interest

The authors declare the following financial interests/personal relationships which may be considered as potential competing interests: YHC, MK and MV report no CoI. HMB has a secondment at HollandPTC and received grants from The Netherlands Organization for Health Research and Development (ZonMw), Medical Delta, the Dutch Healthcare Institute (ZIN) and the CADTH (Canadian Agency for Drugs and Technologies in Health), and an advisory board fee from Pfizer, payments were made to the institute, outside the submitted work and reports to be part of the scientific advisory board of the Dutch Healthcare Institute. CAU has received grants or contracts from Boehringer Ingelheim, Astellas, Celgene, Sanofi, Janssen-Cilag, Bayer, Amgen, Genzyme, Merck, Gilead, Novartis, AstraZeneca, Roche, NIH, and ASCERTAIN, all payments were made to the institute, outside the submitted work. MH reports that Erasmus MC received research grants from Varian Medical Systems a Siemens Healthineers Company, USA, Accuray, USA, Elekta AB, Stockholm Sweden, RaySearch, Stockholm, Sweden, and Siemens Healthineers; HollandPTC received research grants from Varian Medical Systems a Siemens Healthineers Company; received payments paid to Erasmus MC for presentation and support for attending meeting from Accuray, USA outside the submitted work.
